# Olivomycin A Targets Epithelial–Mesenchymal Transition, Apoptosis, and Mitochondrial Quality Control in Renal Cancer Cells

**DOI:** 10.3390/antiox14111348

**Published:** 2025-11-10

**Authors:** Ching-Yu Hsieh, Yih-Farng Liou, Yu-Tung Shih, Alexander S. Tikhomirov, Andrey E. Shchekotikhin, Pin Ju Chueh

**Affiliations:** 1 Graduate Institute of Biomedical Sciences, College of Medicine, National Chung Hsing University, Taichung 402202, Taiwan; a0979287988@gmail.com (C.-Y.H.); d109059004@mail.nchu.edu.tw (Y.-T.S.); 2 Department of Internal Medicine, Feng-Yuan Hospital, Ministry of Health and Welfare, Taichung 42055, Taiwan; liouyihfarngdoc8@gmail.com; 3 Department of Health Administration, Central Taiwan University of Science and Technology, Taichung 406053, Taiwan; 4 Department of Neurosurgery, Jen-Ai Hospital, Taichung 412224, Taiwan; 5 Gause Institute of New Antibiotics, 11 B. Pirogovskaya Street, Moscow 119021, Russia; tikhomirov.chem@gmail.com; 6 Graduate Institute of Chinese Medicine and Drug Development, College of Medicine, National Chung Hsing University, Taichung 402202, Taiwan

**Keywords:** apoptosis, DNA damage, epithelial–mesenchymal transition (EMT), mitochondrial clearance, olivomycin A, renal cell carcinoma (RCC)

## Abstract

Here, we show that the aureolic acid-class antibiotic, olivomycin A, exerts potent anticancer activity in renal cell carcinoma (RCC) by disrupting both cell survival and metastatic programs. In A-498 (wild-type p53) and 786-O (loss-of-function in p53 and PTEN) cells, olivomycin A markedly inhibited migratory capacity and reversed epithelial–mesenchymal transition (EMT), as shown by downregulation of nuclear Snail and the mesenchymal marker N-cadherin and restoration of the epithelial markers, E-cadherin and ZO-1. In parallel, olivomycin A induced apoptosis through distinct p53-dependent mechanisms: In A-498 cells, apoptosis was primarily mediated through the intrinsic pathway, characterized by the upregulation of Puma, Bak, and activation of caspase-9. In 786-O cells, caspase-8 activation and Bid truncation were observed alongside mitochondrial involvement, suggesting possible cross-talk apoptotic cascades. Notably, in p53-mutant 786-O cells, treatment with olivomycin A elicited severe genotoxic stress accompanied by robust DNA damage signaling, excessive reactive oxygen species (ROS) accumulation, and lysosomal activation, culminating in extensive mitochondrial removal. Such changes were weaker in p53-wild-type A-498 cells, suggesting that the altered p53 context sensitizes RCC cells to olivomycin A-mediated mitochondrial quality control mechanisms. Collectively, our findings delineate a multifaceted mechanism whereby olivomycin A coordinates EMT suppression, apoptotic induction, and mitochondrial clearance. Thus, olivomycin A has potential as a therapeutic candidate that can target both survival and metastatic pathways in heterogeneous genetic backgrounds.

## 1. Introduction

The aureolic acid class of antibiotics includes mithramycin, chromomycin A3, and olivomycin A, and was recognized back in the 1960s as having significant antitumor properties [1,2]. Studies in primary cultures derived from human brain tumors revealed that more than 50% of tumor cells were susceptible to these antibiotics [3]. Among the aureolic-class antibiotics, olivomycin A (also known as olivomycin I), produced by *Streptomyces olivoreticuli*, has attracted particular interest due to its intricate aglycone structure, which includes both a disaccharide and a trisaccharide branch. The primary intracellular target of olivomycin A is DNA, specifically GC-rich regions within the minor groove, where it binds through hydrogen interactions between the chromophore of the antibiotic and the 2-amino group of guanine (G4) in the DNA structure [4,5,6]. The consensus 5′-GG-3′ or 5′-GC-3′ binding sequences for olivomycin A are commonly found in the regulatory segments of genes, which also act as potential recognition sites for transcription factors and are associated with many essential biological pathways [7,8,9,10,11,12]. Given the ability of olivomycin A to bind to DNA in these regions, it is unsurprising that this antibiotic can obstruct polymerase function and block subsequent replication and transcription.

Beyond its ability to bind DNA, olivomycin A may also embody its anticancer properties by directly and/or indirectly acting on cellular protein targets. It has been shown to significantly affect a variety of cellular proteins, including heat shock proteins and transcription factors [11,12,13]. Olivomycin A may interfere with the DNA-dependent enzyme topoisomerase I, and can induce cytotoxicity in murine leukemia and human T lymphoblastic cells at nanomolar concentrations [14,15]. A relatively recent study showed that olivomycin A and its derivative, olivamide, inhibit DNA methyltransferase activity to hinder DNA methylation, which is an essential process in epigenetic regulation [16]. In addition, olivomycin A suppresses p53-dependent transcription and promotes apoptosis in human tumor cells [17]. While the tumor suppressor p53 is well-known for its involvement in cell cycle regulation, apoptosis, and DNA repair, knowledge remains limited beyond the context of olivomycin A-inhibited, p53-dependent apoptosis. The ability of olivomycin A to modulate p53 could be crucial for its anticancer effects. However, the detailed molecular mechanisms underlying these effects, particularly the impact of olivomycin A on p53 modulation and downstream signaling, remain inadequately explored. Here, we aimed to address this research gap.

To this end, we employed two human renal cell carcinoma cell lines, A-498 cells expressing wild-type p53 and 786-O cells harboring loss-of-function mutations in both p53 and the phosphatase and tensin homolog (PTEN) genes [18]. We aimed to investigate the mechanism underlying the anticancer activity of olivomycin A in these genetically distinct backgrounds. Our results revealed that the antibiotic triggered apoptosis through p53-dependent mechanisms, activating the intrinsic pathway in A-498 cells and engaging both intrinsic and extrinsic cascades in 786-O cells. Notably, olivomycin A induced more pronounced DNA damage and promoted extensive mitochondrial removal in the p53-mutated background. Together, our findings identify olivomycin A as a promising therapeutic candidate that suppresses EMT and enforces mitochondrial quality control to inhibit the growth of renal cancer cells.

## 2. Materials and Methods

### 2.1. Chemistry

Olivomycin A was produced by *Streptoverticillium cinnamoneum* at the pilot facility of the Gause Institute of New Antibiotics (Moscow, Russia). The compound was purified to 95% purity, as confirmed by reverse-phase HPLC [15].

### 2.2. Cell Culture and Reagents

Anti-Snail, anti-N-cadherin, anti-ZO-1, anti-VCAM-1, anti-FLIP, anti-caspase 8, anti-Bid, anti-Puma, anti-Bak, anti-Bcl-2, anti-caspase 9, anti-PARP, anti-phospho-p53, anti-phospho-Histone H2A.X, anti-PINK1, and anti-Parkin were from Cell Signaling Technology, Inc. (Beverly, MA, USA). The anti-E-cadherin antibody was from Servicebio (Hubei, China), and the anti-cytochrome c antibody was purchased from Santa Cruz Biotechnology, Inc. (Santa Cruz, CA, USA). The anti-β-actin antibody was from Millipore Corp. (Temecula, CA, USA). All other chemicals were from Sigma-Aldrich Corporation (St. Louis, MO, USA). 

The human renal carcinoma cell lines A-498 (BCRC 60241) and 786-O (BCRC 60243) were obtained from the Bioresource Collection and Research Center (BCRC, Hsinchu, Taiwan). A-498 human kidney carcinoma cells (derived from kidney tissues) were cultured in Minimum Essential Medium (MEM) supplemented with 0.1 mM non-essential amino acids and 1.0 mM sodium pyruvate. 786-O human primary renal cell adenocarcinoma cells were cultured in Roswell Park Memorial Institute (RPMI) medium containing 4.5 g/L glucose, 10 mM HEPES, and 1.0 mM sodium pyruvate. All media were supplemented with 10% fetal bovine serum (FBS), 100 U/mL penicillin, and 50 µg/mL streptomycin. Cells were maintained at 37 °C in a humidified incubator with 5% CO_2_ and 95% air.

### 2.3. Wound Healing Assays

Cell migration was evaluated using a two-well culture insert. Inserts were placed in six-well plates, and 100 µL of cell suspension was added to each well at densities of 7.5 × 10^4^ A498 cells or 5.5 × 10^4^ 786-O cells. After incubation at 37 °C with 5% CO_2_ for 16 h to allow attachment, 1 mL of fresh medium was added to the surrounding well area, and inserts were carefully removed to create a cell-free gap. Images of the initial wound area (0 h) were acquired immediately after the insert removal. Cells were then treated with olivomycin A and incubated for 8 h under the same culture conditions, followed by image acquisition to document cell migration into the wound area. Wound closure was quantified using ImageJ software (version 1.8.0; National Institutes of Health, Bethesda, MD, USA), with the wound area at 0 h defined as 100%. The remaining wound area after 8 h was expressed as a percentage, with smaller residual wound areas indicating greater migratory activity.

### 2.4. Transwell Assay

The integrity of the transwell inserts was verified by adding 300 µL PBS the day before the experiment to check for leakage. After 24 h of Olivomycin A treatment, 2 × 10^4^ cells suspended in 200 µL serum-free medium were seeded into the upper chamber and allowed to adhere to the membrane for 20 min at 37 °C in 5% CO_2_. The lower chamber was then filled with 600 µL medium containing 10% FBS as a chemoattractant, and cells were incubated for an additional 24 h. Following incubation, the medium was removed from both chambers, and cells were washed twice with PBS and fixed with 100% methanol (200 µL in the upper chamber, 600 µL in the lower chamber) for 10 min. After two washes with PBS, cells were stained overnight at room temperature with Giemsa solution diluted 1:10 in ddH_2_O (200 µL upper chamber, 600 µL lower chamber). The next day, the stain was removed, membranes were washed with ddH_2_O and soaked for ~2 h, air-dried, and imaged under a light microscope. Migrated cells were quantified using ImageJ software (version 1.8.0; National Institutes of Health, Bethesda, MD, USA).

### 2.5. Cell Impedance Measurements

Cell proliferation was monitored using the xCELLigence Real-Time Cell Analysis (RTCA) (Roche, Mannheim, Germany) according to the manufacturer’s instructions. Experimental parameters, including E-Plate identification, cell line, seeding density, and drug treatment conditions, were first configured using the standard RTCA Software Pro (version 2.6.1) provided by the manufacturer. Each well of the E-Plate was filled with 75 µL of fresh culture medium, avoiding bubble formation, and the plate was placed in the instrument for background impedance measurement. Subsequently, 75 µL of culture medium containing 4000 786-O cells was added to each well (final volume, 150 µL), taking care to prevent bubbles and to avoid disturbing the microelectrode surface. After gentle mixing, the E-Plate was inserted into the RTCA instrument and incubated at 37 °C with 5% CO_2_ for 16 h to allow cell attachment. Drug treatment was carried out by pausing the RTCA software (version 2.6.1) and carefully aspirating 75 µL of supernatant from each well without disturbing adherent cells. An equal volume (75 µL) of fresh medium containing olivomycin A at the indicated concentrations was then added. Plates were gently mixed, returned to the RTCA instrument, and continuously monitored for impedance-based cell index measurements.

### 2.6. Colony-Forming Assay

Cells were seeded into six-well plates at a density of 1200 A498 cells or 500 786-O cells per well in 2 mL of fresh culture medium and incubated at 37 °C in 5% CO_2_ for 48 h to allow attachment. Afterward, the medium was replaced with 3 mL of fresh culture medium containing olivomycin A at the indicated concentrations, and cells were cultured for an additional 9 days with medium changes every 3 days. At the end of treatment, colonies were washed twice with PBS, fixed with 100% methanol for 10 min, and stained with 0.05% crystal violet for 1–3 min. Excess dye was removed by rinsing with ddH_2_O until the background was clear, and plates were air-dried before scanning. Colony formation was quantified using ImageJ software (version 1.8.0; National Institutes of Health, Bethesda, MD, USA), and results were expressed as the relative colony area per well.

### 2.7. Apoptosis Determination

Cells were seeded in six-well plates at a density of 3 × 10^5^ A498 cells or 2.5 × 10^5^ 786-O cells per well in 2 mL of fresh culture medium and incubated at 37 °C in 5% CO_2_ for 16 h to allow attachment. Cells were then treated with olivomycin A at the indicated concentrations, while 0.03% H_2_O_2_ was used as a positive control (PC), and incubated for 24 h. After treatment, culture supernatants were collected, cells were washed once with 1 mL PBS, detached with trypsin, and harvested by centrifugation (1000 rpm, 1 min). Pellets were washed with PBS, transferred to 1.5 mL tubes, and centrifuged again (2000 rpm, 3 min). PC samples were divided into three groups for Annexin V single staining, PI single staining, or Annexin V/PI double staining, while all other groups were subjected to double staining. For staining, 10× Annexin V binding buffer was diluted to 1×, and dyes were diluted 1:1000 in 1× buffer. Cells were resuspended in 500 µL staining solution and incubated for 20 min at room temperature in the dark. After gentle resuspension and filtration through a cell strainer to avoid clogging, samples were analyzed on a Beckman Coulter CytoFLEX LX flow cytometer (Brea, CA, USA) using Annexin V-FITC and propidium iodide (PI) channels. Unstained cells served as negative controls to define gating, followed by compensation using single-stained samples. Apoptosis was quantified as the percentage of Annexin V-positive and/or PI-positive cells using the BD Pharmingen FITC Annexin V Apoptosis Detection Kit (BD Biosciences, San Jose, CA, USA) according to the manufacturer’s instructions.

### 2.8. Measurement of Cellular Reactive Oxygen Species (ROS)

Oxidative stress was assessed by detecting intracellular hydrogen peroxide using 5-(and-6)-carboxy-2′,7′-dichlorodihydrofluorescein diacetate (carboxy-H_2_DCFDA). This nonpolar, cell-permeable dye is hydrolyzed by intracellular esterases to form the nonfluorescent H_2_-DCF, which is subsequently oxidized by peroxides to yield the highly fluorescent DCF. For staining, sterilized coverslips were placed into 24-well plates containing 1 mL of fresh medium, and cells were seeded at a density of 2.5 × 10^4^ cells per well. After overnight incubation at 37 °C with 5% CO_2_ to allow attachment, cells were washed once with 1 mL of PBS and once with 0.5 mL of 1× buffer (diluted in ddH_2_O), followed by incubation with 0.5 mL of 1× buffer containing the indicated concentrations of olivomycin A. After 15 min of drug exposure, cells were stained with 4 µM DCFDA for 45 min at 37 °C in 5% CO_2_ under light-protected conditions. Cells were washed twice with 1× buffer before imaging, and coverslips were inverted onto glass slides for fluorescence microscopy. Fluorescence images were acquired using an excitation/emission setting of 488/535 nm.

### 2.9. Immunofluorescence Staining

Cells were seeded on sterilized coverslips placed in 24-well plates (2.2 × 10^4^ A498 cells or 2.0 × 10^4^ 786-O cells per well) and cultured overnight at 37 °C with 5% CO_2_ to allow attachment, followed by treatment with olivomycin A at the indicated concentrations. For live-cell staining, cells were incubated with 20 nM MitoTracker Green for 30 min or 50 nM LysoTracker Red for 5 min at 37 °C in the dark. After washing with PBS, cells were fixed with 4% paraformaldehyde for 15 min, permeabilized with 0.1% Triton X-100 for 10 min, and blocked with buffer containing 10% FBS and 3% BSA for 1 h at room temperature. Primary antibodies, diluted in blocking buffer according to manufacturer recommendations, were applied for 1 h, followed by fluorophore-conjugated secondary antibodies (1:500) for an additional 1 h. Nuclei were counterstained with 1 µg/mL DAPI for 5 min. Coverslips were mounted on glass slides with mounting medium, edges sealed with nail polish, and stored at 4 °C in the dark until confocal fluorescence microscopy.

### 2.10. Western Blot Analysis

Cell extracts were prepared in lysis buffer containing 20 mM Tris-HCl (pH 7.4), 100 mM NaCl, 5 mM EDTA, 2 mM phenylmethylsulfonyl fluoride (PMSF), 10 ng/mL leupeptin, and 10 μg/mL aprotinin. Protein concentrations were determined, and equal amounts of protein (40 µg per sample) were separated by SDS-PAGE and transferred onto nitrocellulose membranes (Schleicher & Schuell, Keene, NH, USA). Membranes were blocked with nonfat milk solution for 30 min, washed, and incubated with primary antibody. After washing with Tris-buffered saline containing 0.1% Tween 20 (TBS) to remove unbound antibody, membranes were incubated with horseradish peroxidase (HRP)-conjugated secondary antibody for 2 h. Bound antibodies were visualized using enhanced chemiluminescence (ECL) detection reagents (Amersham Biosciences, Piscataway, NJ, USA).

### 2.11. Statistics

All data are presented as the mean ± standard deviation (SD) from at least three independent experiments. Comparisons between groups were performed using one-way analysis of variance (ANOVA) followed by an appropriate post hoc test. Differences were considered statistically significant at *p* < 0.05.

## 3. Results

### 3.1. Olivomycin a Induces Antiproliferative Effects and Cellular Senescence in Renal Cancer Cells

**Figure 1:**
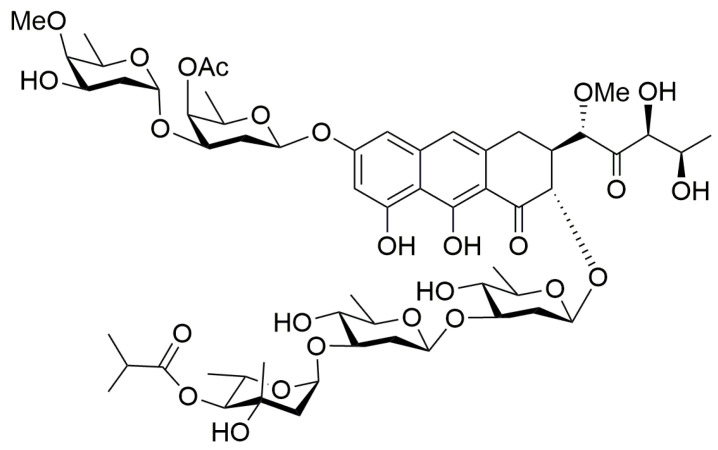
Structure of olivomycin A.

The utilized olivomycin A was produced by *Streptoverticillum cinnamomeum* at the pilot production line of the Gause Institute of New Antibiotics in Moscow (Figure 1).

**Figure 2:**
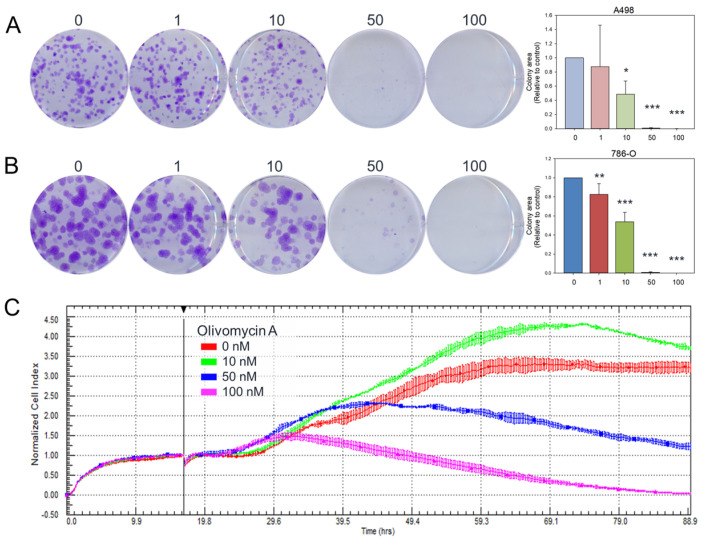
Effects of olivomycin A on colony formation and proliferation in renal cancer cells. (**A**,**B**) Representative images and quantification of colony formation assays showing reduced clonogenic survival in A-498 (**A**) and 786-O (**B**) cells following treatment with olivomycin A. Colony numbers were determined and documented. Data are presented as mean ± SD from at least three independent experiments. There was a significant reduction in colony numbers in olivomycin A-treated cells compared with untreated controls (* *p* < 0.05, ** *p* < 0.01, *** *p* < 0.001 vs. control). (**C**) Cell proliferation was dynamically monitored using the xCELLigence system in 786-O cells, demonstrating concentration-dependent growth inhibition.

To explore whether olivomycin A exhibits anticancer activities against kidney cancer cells and the potential impact of p53 in the system, we employed two human renal cell carcinoma cell lines: A-498 cells, which carry wild-type p53, and 786-O cells, which harbor mutant p53 and PTEN genes. First, we assessed the long-term effect of olivomycin A on renal cancer cell proliferation using a colony-forming assay. The results showed that the antibiotic markedly reduced the colony numbers in both cell lines, with a significant inhibitory effect observed starting at 10 nM for A-498 cells (Figure 2A) and 1 nM for 786-O cells (Figure 2B). The apparent antiproliferative effect was further validated by performing real-time cell impedance measurements with an xCELLigence system, which continuously tracks cell growth based on electronic impedance. Consistent with the results of our colony formation assays, the cell impedance measurements showed that olivomycin A markedly attenuated the proliferation of 786-O cells. At 10 nM, a transient proliferative effect was observed, but this was overridden at higher concentrations, with robust growth inhibition evident at 50 and 100 nM in these p53-mutated cells (Figure 2C). In contrast, the p53-wild-type A-498 cells exhibited nonlinear impedance changes and fluctuating cell index values even in the absence of olivomycin A, likely due to partial detachment from the electrode surface. Because these fluctuations were not reproducible across independent experiments, a reliable olivomycin A-responsive proliferation profile could not be established for this cell line.

### 3.2. Olivomycin a Suppresses Renal Cancer Cell Migration by Modulating EMT Markers

**Figure 3:**
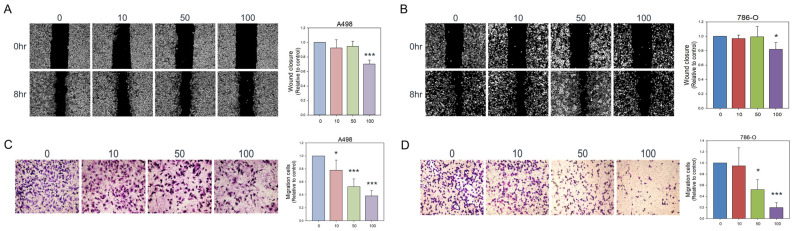
Inhibitory effects of olivomycin A on cell migration assessed by wound healing assays and transwell chamber assays. (**A**,**B**) The cell monolayer was scratched with a pipette tip and treated with different concentrations of olivomycin A or vesicles as a control in A-498 (**A**) and 786-O (**B**) cells. Wound closure was examined at 0 and 8 h after scratching using inverted light microscopy. Representative images from three independent experiments are shown. Quantitative analysis of wound closures is presented in the histogram. Values represent means ± SE from three independent experiments performed in triplicate (* *p* < 0.05, *** *p* < 0.001 vs. control). (**C**,**D**) Cell migration was further assessed by transwell chamber assays. Migrated cells were fixed, stained, and counted in A-498 (**C**) and 786-O (**D**) cells. Quantitative analysis of migrated cells is shown in the histogram. Values represent means ± SE from three independent experiments performed in triplicate (* *p* <0.05, *** *p* <0.001 vs. control).

Having established that olivomycin A exerts an antiproliferative effect on renal cancer cells, we next investigated whether it also impacts their migratory capacity. Wound healing assays revealed that 100 nM olivomycin A significantly suppressed migration in both A-498 cells (Figure 3A) and 786-O cells (Figure 3B). The time-course data with multiple time points are provided in Supplementary Figure S1. The results consistently showed that olivomycin A markedly delayed wound closure in both cell lines compared with controls. This inhibitory effect was further validated using Boyden transwell assays, which showed pronounced migration suppression at concentrations as low as 10 nM for A-498 cells (Figure 3C) and 50 nM for 786-O cells (Figure 3D).

**Figure 4:**
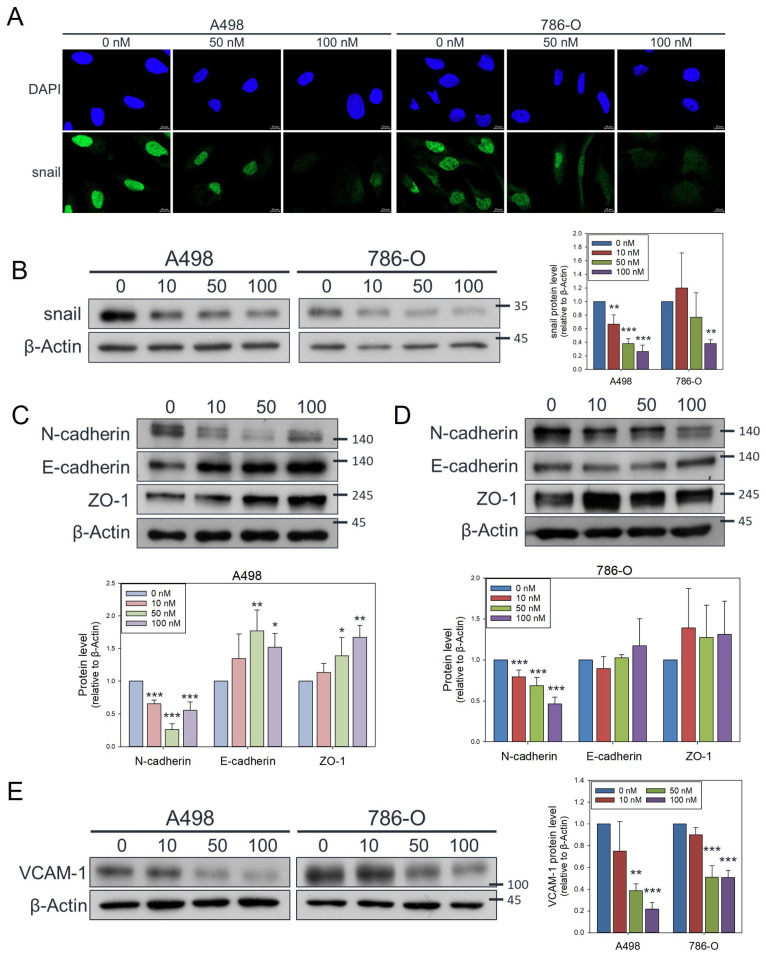
Attenuated effects of olivomycin A on Snail, epithelial–mesenchymal transition markers, and VCAM-1 in renal cancer cells. (**A**) Confocal microscopy of nuclear Snail (green) and DAPI (blue) staining. Cells were treated with olivomycin A and a vehicle control and subjected to immunofluorescence analysis. Representative confocal images demonstrate reduced nuclear localization of Snail in olivomycin A–treated cells compared with controls. (**B**) Western blot analysis showing that olivomycin A markedly attenuated Snail protein expression in both A-498 and 786-O cells. (**C**) In A-498 cells, olivomycin A significantly reduced N-cadherin while upregulating E-cadherin and ZO-1 expression. (**D**) In 789-O cells, olivomycin A significantly downregulated N-cadherin and modestly increased E-cadherin and ZO-1 expression. (**E**) Olivomycin A also significantly reduced VCAM-1 expression in both A-498 and 798-O cells. Cell lysates were resolved by SDS-PAGE and analyzed by Western blotting, with β-actin used as a loading control. Representative blots are shown. Quantitative data represent means ± SE from three independent experiments performed in triplicate (* *p* < 0.05, ** *p* < 0.01, *** *p* < 0.001 vs. control).

Mechanistically, this reduction in migratory ability was associated with decreases in the nuclear localization (Figure 4A) and total protein levels of the transcription factor Snail (Figure 4B), which is a key driver of EMT. In A498 cells, olivomycin A significantly downregulated the mesenchymal marker, N-cadherin, while upregulating the epithelial markers, E-cadherin, and ZO-1 (Figure 4C). 786-O cells showed more modest induction of E-cadherin and ZO-1, but a notable suppression of N-cadherin similar to that seen in A-498 cells (Figure 4D). Because EMT not only enhances motility but also facilitates tumor-endothelial interactions critical for intravasation, we next examined the adhesion molecule vascular cell adhesion molecule-1 (VCAM-1). The expression of VCAM-1 was notably decreased in both cell lines following treatment with 50 nM olivomycin A (Figure 4E), suggesting that olivomycin A-treated cells may exhibit impaired adhesion capacity during intravasation. Collectively, these results demonstrate that olivomycin A suppresses renal cancer cell migration by modulating EMT-related proteins—downregulating Snail and N-cadherin while enhancing E-cadherin and ZO-1—thereby reinforcing epithelial characteristics and limiting invasive potential.

### 3.3. Olivomycin a Induces Cell Line-Specific Apoptotic Pathways in Renal Cancer Cells

**Figure 5:**
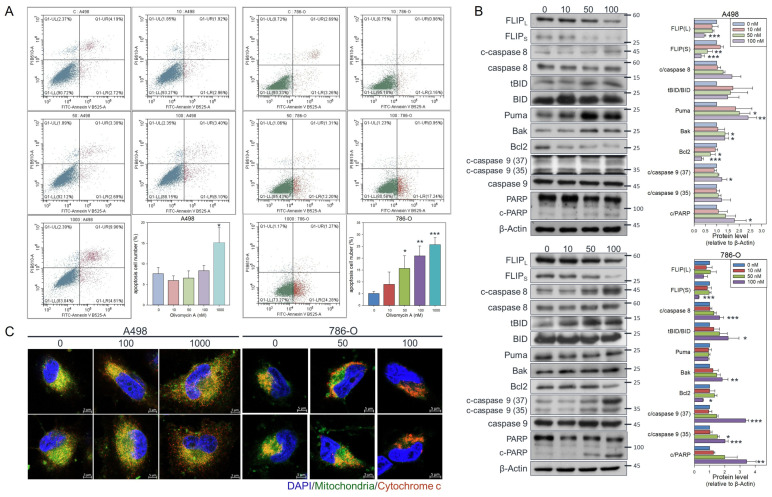
Olivomycin A induces apoptosis in renal cancer cells. (**A**) Cells were treated with olivomycin A or vesicle control for 24 h, and the percentage of apoptotic cells was determined by flow cytometry. Results are presented as the percentage of apoptotic cells; values represented mean ± SE from three independent experiments performed in triplicate (* *p* <0.05, ** *p* <0.01, *** *p* <0.001 vs. controls). (**B**) Western blot analysis showing that olivomycin A markedly increased pro-apoptotic markers while reducing anti-apoptotic proteins in A-498 (top) and 786-O (bottom) cells. Cell lysates were resolved by SDS-PAGE and analyzed by Western blotting, with β-actin used as a loading control. Representative blots are shown. Quantitative data represent means ± SE from three independent experiments performed in triplicate (* *p* < 0.05, ** *p* < 0.01, *** *p* < 0.001 vs. control). (**C**) Confocal microscopy of DAPI (blue), mitochondria (green), and cytochrome c (red) staining. Cells were treated with olivomycin A and a vehicle control and subjected to immunofluorescence analysis. Representative images show increased cytochrome c release from mitochondria into the cytoplasm in olivomycin A-treated cells compared with controls.

To further elucidate the molecular events underlying olivomycin A-induced cell proliferation inhibition, we explored the cell death pathway and related signaling mechanisms. Since olivomycin A has been reported to induce apoptosis in cancer cells [13,15,17], we employed Annexin V staining and flow cytometry to analyze the apoptotic populations in our experimental system. Our results showed that olivomycin A effectively induced apoptosis at 1 μM in A-498 and at 50 nM in 786-O cells (Figure 5A). To clarify the apoptotic pathways contributing to this olivomycin A-mediated growth inhibition, we conducted protein expression analysis. In A498 cells, both isoforms of the antiapoptotic protein, FLIP, were significantly downregulated by olivomycin A; however, this was not accompanied by caspase-8 activation or Bid truncation. Instead, we observed upregulation of Puma, Bak, and activated caspase-9 (Figure 5B top panel). These results indicate that olivomycin A activated the intrinsic apoptotic pathway in A-498 cells. By contrast, we observed downregulation of FLIP and upregulation of activated caspase-8, truncated Bid, Puma, and Bak, accompanied by a reduction in the antiapoptotic protein, Bcl-2, as well as caspase-9 activation and PARP cleavage (Figure 5B bottom panel). We further examined cytochrome c localization and found that, consistent with the flow cytometry data (Figure 5A), olivomycin A promoted its release from mitochondria into cytoplasm, with a more pronounced effect in 786-O than that in A498 cells (Figure 5C). Collectively, these findings indicate that olivomycin A triggers apoptosis in renal cancer cells primarily through mitochondrial (intrinsic) signaling, with caspase-activation suggesting potential cross-talk between apoptosis cascades rather than definitive involvement of a receptor-mediated extrinsic pathway.

### 3.4. Olivomycin a Induces Mitochondrial Stress and Mitophagy Through PINK1 Signaling in Renal Cancer Cells with Mutant P53

**Figure 6:**
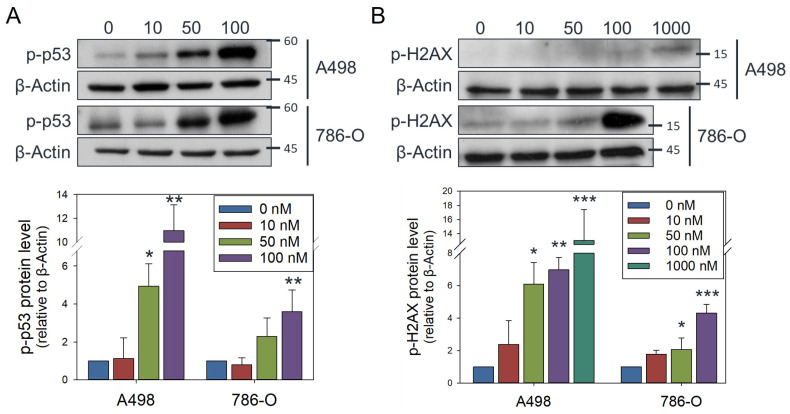
Olivomycin A induces DNA damage signaling in renal cancer cells. (**A**,**B**) Western blot analysis showing that olivomycin A provoked expression of phosphorylated p53 (**A**) and H2AX (**B**). Cell lysates were resolved by SDS-PAGE and probed by the indicated antibodies, with β-actin used as a loading control. Representative blots are shown. Quantitative data represent means ± SE from three independent experiments performed in triplicate (* *p* < 0.05, ** *p* < 0.01, *** *p* < 0.001 vs. control).

Given the significant mitochondrial loss observed in 786-O cells exposed to 100 nM olivomycin A–which harbor mutated p53—but not A-498 cells carrying wild-type p53 (Figure 5C), we sought to determine whether p53 functionality contributes to these differential responses. Total p53 expression was elevated by olivomycin A in both cell lines, and a parallel increase in phosphorylated p53 was detected (Figure 6A). Because total p53 levels also rose, the apparent phosphorylation signal likely reflects enhanced overall p53 accumulation rather than selective phosphorylation. In contrast, phosphorylated H2AX was markedly upregulated only in 786-O cells (Figure 6B), indicating that olivomycin A induced a stronger DNA-damage response in the mutant p53 background.

**Figure 7:**
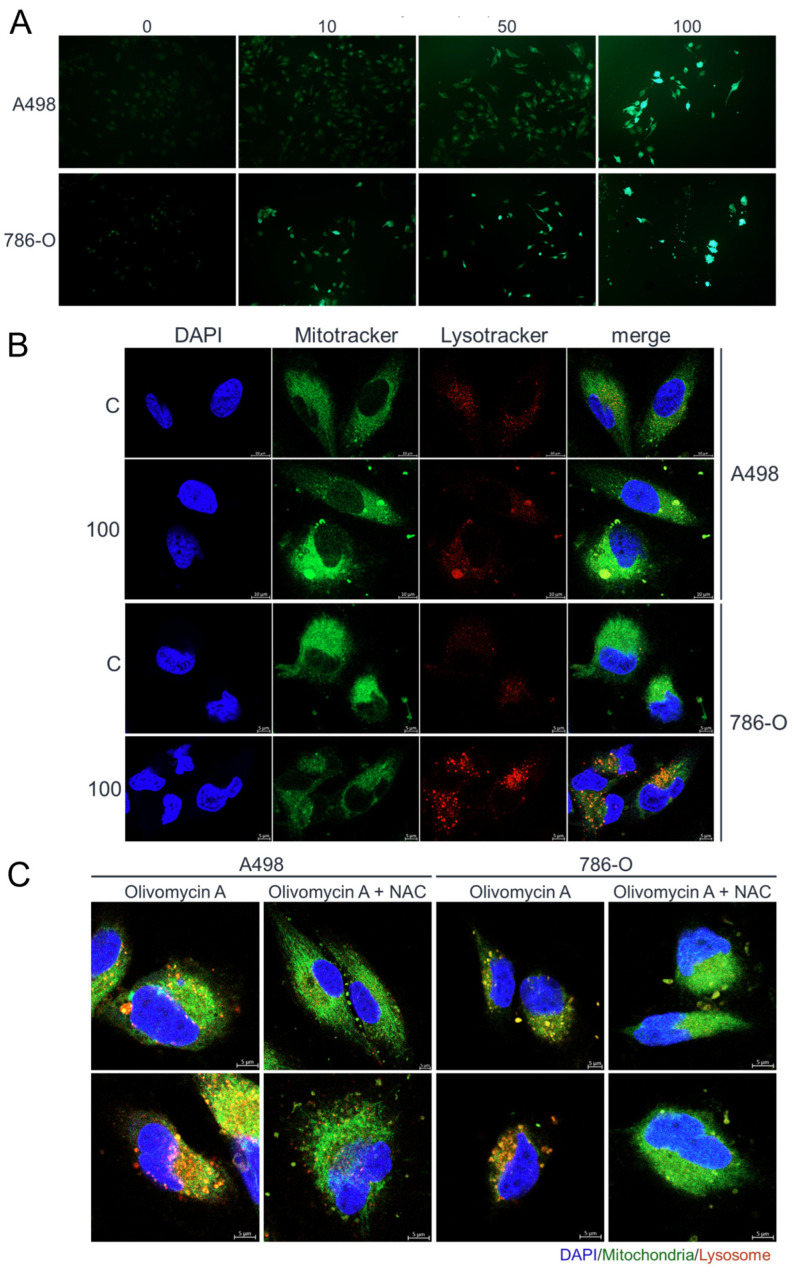
Olivomycin A induces oxidative stress and lysosomal activity in renal cancer cells, effects reversed by NAC. (**A**) Fluorescent microscopy of DCFDA staining showing elevated ROS levels in cells treated with olivomycin A compared with vehicle control. (**B**) Confocal microscopy of DAPI (blue), MitoTracker (green), and LysoTracker (red) staining showing that olivomycin A treatment increased lysosomal activity and mitochondrial stress in 786-O cells, but not in A486 cells, relative to controls. (**C**) Confocal microscopy of DAPI (blue), MitoTracker (green), and LysoTracker (red) staining showing that NAC co-treatment attenuated olivomycin A-induced lysosomal activity and mitochondrial stress. Representative images are shown.

This indicated that 50 nM olivomycin A treatment triggered severe DNA damage in the mutant, but not wild-type, p53 background. This extensive DNA damage was also accompanied by a more pronounced increase in overall reactive oxygen species (ROS) levels, as measured by the general ROS probe carboxyl-H_2_DCFDA, following treatment with 10 nM olivomycin A in 786-O cells compared with A-498 cells (Figure 7A). Consistently, Lysotracker staining revealed that olivomycin A treatment substantially increased lysosomal activity in 786-O cells, whereas the effect was comparatively modest in A-498 cells (Figure 7B). This suggests activation of the autophagic-lysosomal pathway, which may contribute to the reduced mitochondrial content observed in olivomycin A-treated p53-mutant cells, as evidenced by a marked reduction in green fluorescence intensity (Figure 7B). To further examine the relationship between ROS and mitochondrial depletion, we employed the antioxidant N-acetylcysteine (NAC). NAC pretreatment effectively reversed olivomycin A-induced lysosomal activation in both A-498 and 786-O cells (Figure 7C). Importantly, inhibition of ROS markedly prevented olivomycin A-induced mitochondrial clearance in 786-O cells, indicating that ROS accumulation plays a critical role in mediating mitochondrial stress and removal in the p53-mutant background. Together, these results support that olivomycin A induces oxidative and mitochondrial stress preferentially in p53-mutant RCC cells.

**Figure 8:**
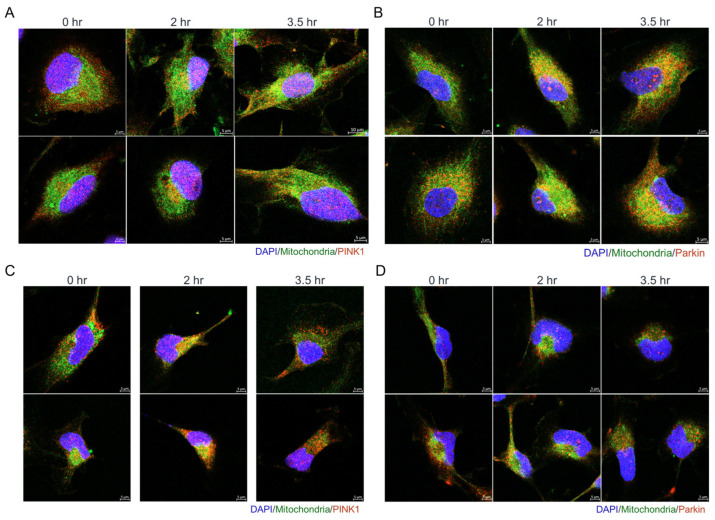
Olivomycin A induces colocalization of mitochondria with PINK1 and Parkin in renal cancer cells. (**A**,**C**) Confocal microscopy of DAPI (blue), MitoTracker (green), and PINK1 (red) staining showing that olivomycin A treatment increases the localization of PINK1 to mitochondria modestly in A-498 cells (**A**), and prominently in 786-O cells (**C**), relative to controls. (**B**,**D**) Confocal microscopy of DAPI (blue), MitoTracker (green), and Parkin (red) staining showing that olivomycin A treatment increases the localization of Parkin to mitochondria in A-498 cells (**B**) but not in 786-O cells (**D**), relative to controls. Representative images are shown.

These observations prompted us to speculate that, in olivomycin A-treated renal cancer cells, particularly in the p53-mutant background, selective autophagic removal of damaged mitochondria may occur as part of the cellular response to oxidative and genotoxic stress. Given that the PTEN-induced kinase 1 (PINK1)-Parkin pathway plays an essential role in ROS-mediated mitochondrial quality control [19], we examined the subcellular localizations of PINK1 and Parkin by immunofluorescence staining of olivomycin A-treated cells. In A-498 cells, exposure to 100 nM olivomycin A resulted in modest colocalization of mitochondria with PINK1 at 3.5 h post-treatment (Figure 8A) and with Parkin at 2 h post-treatment (Figure 8B). In contrast, in 786-O cells, which harbor mutations in p53, PTEN, and PINK1, PINK1 accumulation on mitochondria was evident at early as 2 h, followed by a marked reduction in mitochondrial content at 3.5 h (Figure 8C). However, Parkin recruitment to mitochondria was less pronounced in 786-O cells (Figure 8D), possibly reflecting impairing signaling due to mutant PINK1 in this background. Together, these results suggest that olivomycin A promotes PINK1-associated mitochondrial remodeling and clearance in renal cancer cells, particularly in those with mutant p53, although the precise degradation pathway remains to be determined.

## 4. Discussion

Kidney cancer encompasses several histologically and molecularly distinct subtypes and ranks as the 14th most common malignancy worldwide, with approximately 180,000 deaths reported in 2020 [20,21,22]. Among the subtypes of kidney cancer, renal cell carcinoma (RCC) is the predominant form, accounting for nearly 90% of all renal malignancies. Genomic studies have identified at least 11 recurrent mutated genes implicated in RCC pathogenesis, including those encoding Von Hippel-Lindau (VHL) tumor suppressor and PTEN [23,24]. Despite advances in surgical resection, targeted therapies, and immune checkpoint inhibitors, the current treatment options for RCC remain limited by both intrinsic and acquired resistance, and durable responses are achieved in only a fraction of patients. This therapeutic challenge underscores the need for agents with novel action mechanisms.

Using two representative RCC cell lines, we herein show that olivomycin A, an aureolic acid-class antibiotic, exerts multifaceted anticancer effects by simultaneously impairing migration capacity and inducing apoptosis and oxidative stress-associated mitochondrial clearance. These multiple actions are clinically significant, given that RCC cells are notoriously resistant to apoptosis and prone to metastasis, both of which contribute to the poor prognosis of this disease [25,26]. By targeting both survival and metastatic programs, olivomycin A may offer a therapeutic advantage over agents that act on a single pathway, particularly in tumors harboring p53, VHL, and PTEN mutations, which constitute a subset of aggressive RCC subtypes [27,28,29,30]. Interestingly, the cellular response to olivomycin A was strongly influenced by p53 status. In p53-wild-type A-498 cells, apoptosis was primarily mediated via the intrinsic pathway, as reflected by upregulation of Puma, Bak, and active caspase-9. In contrast, 786-O cells harboring concurrent mutations in p53 and PTEN displayed additional activation of caspase-8 and Bid truncation, together with FLIP suppression, suggesting potential cross-talk between apoptotic cascades rather than direct engagement of the death receptor-mediated extrinsic pathway. Beyond its role in restraining oncogenic signaling, PTEN also modulates p53 stability and activity through phosphorylation-dependent mechanisms [31], while p53 itself enhances PTEN transcription [32,33,34]. Disruption of this reciprocal tumor-suppressive axis, as in 786-O cells, may alter apoptotic sensitivity and render cells more vulnerable to agents that impose both DNA damage and oxidative stress.

A notable observation was the preferential induction of mitochondrial loss and lysosomal activation in 786-O cells following olivomycin A treatment. Increasing evidence suggests that *p53* and PTEN cooperatively regulate mitochondrial quality control through ROS- and stress-dependent pathways. Under genotoxic stress, selective removal of damaged mitochondria acts as a compensatory mechanism to maintain mitochondrial integrity and redox balance [35,36]. Although direct evidence was not obtained in this study, selective autophagic removal of damaged mitochondria (mitophagy) could play a role in this process. p53 exerts dual and context-dependent effects: Nuclear p53 can repress the transcription of stress-response genes, such as that encoding BNIP3 [37], which mediates receptor-driven mitophagy, whereas cytosolic p53 directly binds Parkin and prevents its mitochondrial translocation, to inhibit canonical PINK1/Parkin-mediated mitophagy [38,39]. In parallel, PTEN, particularly its PTENα isoform, facilitates Parkin recruitment to damaged mitochondria, thereby promoting PINK1/Parkin-dependent mitophagy and maintaining mitochondrial integrity [40,41]. Loss of PTEN disrupts this process, leading to defective mitochondrial turnover and exacerbated ROS accumulation. When combined with *p53* inactivation, this dual deficiency can synergistically impair mitochondrial turnover and redox regulation, thereby sensitizing cells to oxidative and genotoxic insults.

Consistent with these mechanisms, our data show that olivomycin A treatment in p53/PTEN-deficient 786-O cells induced severe DNA damage and ROS accumulation, accompanied by mitochondrial collapse and elevated lysosomal activity, which are hallmarks of mitochondrial stress-induced clearance. Because total intracellular ROS were measured using the general probe carboxy-H_2_DCFDA, the precise mitochondrial contribution remains undetermined. Nonetheless, these findings suggest that excessive ROS generation, coupled with impaired apoptotic control and defective mitochondrial turnover, may represent a therapeutically exploitable vulnerability through which olivomycin A selectively overwhelms mitochondrial quality-control mechanisms in aggressive RCC. Future work will employ mitochondria-targeted ROS assays (e.g., MitoSOX Red) and antioxidant rescue experiments to confirm this causal relationship.

In addition to the dysregulation of mitochondrial quality control, the enhanced DNA damage observed in p53-mutated RCC cells likely reflects their compromised ability to activate canonical p53-dependent DNA damage response and repair pathways. In p53-wild-type cells, olivomycin A–induced oxidative and replication stress can activate p53 signaling, leading to transcriptional induction of *CDKN1A* (p21), *PUMA*, and *GADD45*, which coordinate cell-cycle arrest and DNA repair [42,43,44]. Conversely, loss or mutation of p53 abrogates these checkpoints, resulting in persistent ROS accumulation, defective repair of DNA strand breaks, and amplification of damage signaling [45,46]. This mechanism aligns with prior observations that p53-defective cancer cells exhibit heightened susceptibility to genotoxic and oxidative stressors [47,48]. Although the present study did not directly examine cell-cycle responses to olivomycin A, this will be the focus of future investigations aimed at defining the p53-dependent checkpoint control in RCC.

This study provides mechanistic insights into how olivomycin A may perturb oxidative and lysosomal homeostasis in renal cancer cells, yet several important aspects warrant further investigation. Similarly to other aureolic acid antibiotics, olivomycin A appears to possess a narrow therapeutic window and potential hepatotoxicity, which may limit its systemic applicability. Although phosphorylated *p53* levels increased following olivomycin A exposure, total *p53* levels also rose, suggesting that the apparent phosphorylation likely reflects overall protein accumulation rather than selective activation. Likewise, while caspase-8 activation and Bid truncation suggest potential cross-talk with the extrinsic apoptotic pathway in 786-O cells, receptor–ligand interactions remain to be determined. In addition, the observed mitochondrial fragmentation and lysosomal activation are consistent with enhanced mitochondrial turnover but provide only indirect evidence of mitophagy. Future studies will include assessment of LC3-I/II and p62/SQSTM1 dynamics, mitochondrial colocalization with LC3 or LAMP1, and pharmacologic modulation of autophagy to clarify whether olivomycin A-induced autophagy is protective or cytotoxic. Similarly, although our findings imply oxidative stress involvement, direct quantification of mitochondrial ROS was not performed. Subsequent experiments using MitoSOX Red and antioxidant rescue assays will help determine whether ROS generation contributes causally to DNA damage and apoptosis. Finally, because the current findings are limited to in vitro and ex vivo models, further in vivo studies using xenograft or orthotopic tumor systems will be essential to evaluate therapeutic efficacy and safety. Collectively, these revisions ensure that our interpretations remain appropriately evidence-based, and we have tempered language throughout the manuscript to align conclusions with the data while outlining clear directions for future investigation.

## 5. Conclusions

Collectively, our findings show that the aureolic acid-class antibiotic olivomycin A exerts multifaceted anticancer activity in RCC cells in vitro by suppressing cell migration, triggering apoptosis, and disrupting mitochondrial quality control. These effects are strongly influenced by the mutational status of p53 and PTEN, two pivotal tumor suppressors that together govern apoptotic signaling and mitochondrial homeostasis. In RCC cells harboring mutant versions of p53 and PTEN, the interplay of excessive ROS generation, impaired mitochondrial quality control, and deregulated apoptosis creates a unique vulnerability that can be exploited by agents such as olivomycin A. Thus, targeting the p53/PTEN-mitochondrial clearance axis may represent a promising therapeutic strategy for addressing aggressive RCC subtypes that are refractory to current treatments.

## Data Availability

The original contributions presented in this study are included in the article/Supplementary Material. Further inquiries can be directed to the corresponding author(s).

## Supplementary Materials

The following supporting information can be downloaded at: https://www.mdpi.com/article/10.3390/antiox14111348/s1, Supplementary Figure S1: (A,B) The cell monolayer was scratched with a pipette tip and treated with different concentrations of olivomycin A or vesicles as a control in A-498 (A) and 786-O (B) cells. Wound closure was examined at 0, 2, 4, 6, and 8 h after scratching using inverted light microscopy. Images from three independent experiments are shown.

## Abbreviations

The following abbreviations are used in this manuscript:

EMT	Epithelial–mesenchymal transition
NAC	N-acetylcysteine
PINK1	PTEN-induced kinase 1
PTEN	Phosphatase and tensin homolog
ROS	Reactive oxygen species
RCC	Renal cell carcinoma
VHL	Von Hippel-Lindau tumor suppressor
