# Supplementary material for: Olivomycin A Targets Epithelial–Mesenchymal Transition, Apoptosis, and Mitochondrial Quality Control in Renal Cancer Cells
Source: Antioxidants (Basel). 2025 Nov 10;14(11):1348. doi: 10.3390/antiox14111348 (PMC12649244; doi:10.3390/antiox14111348)
Supplement: Supplementary file 1 [file antioxidants-14-01348-s001.zip › antioxidants-3909748-supplementary.pdf]

Supplementary Figure S1

A. A498 cells

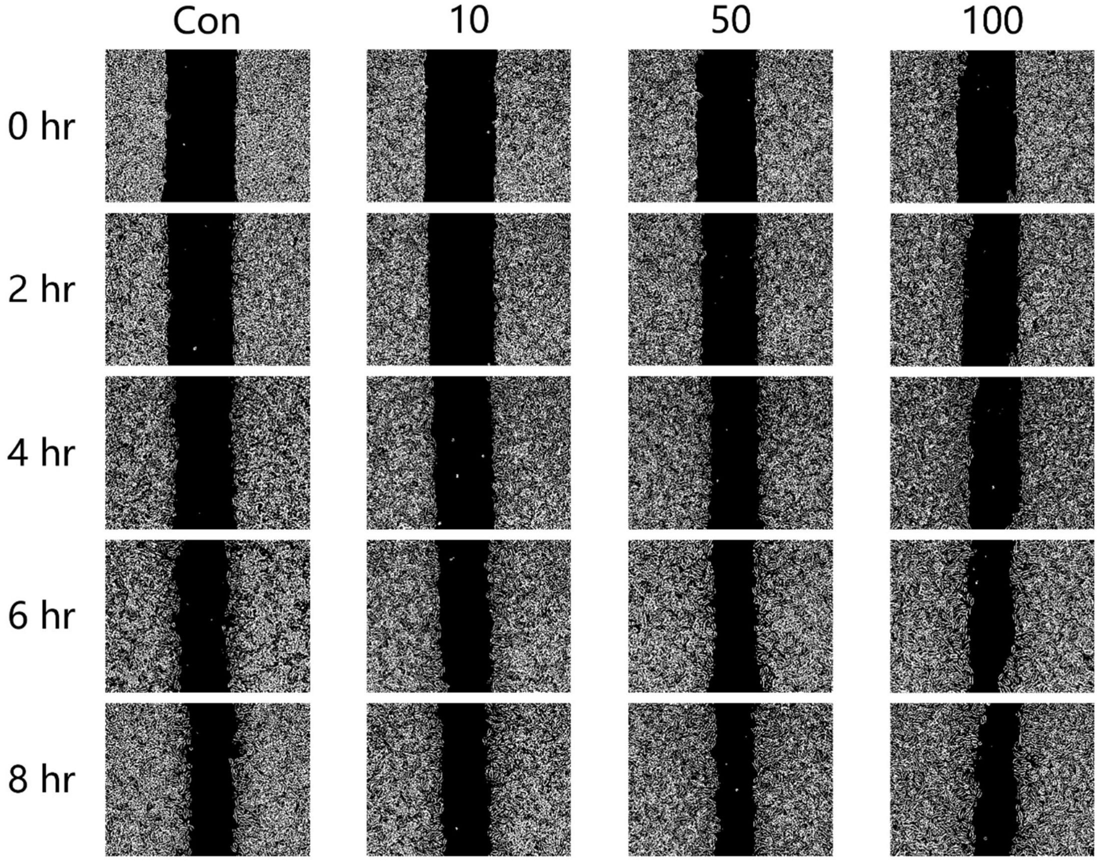

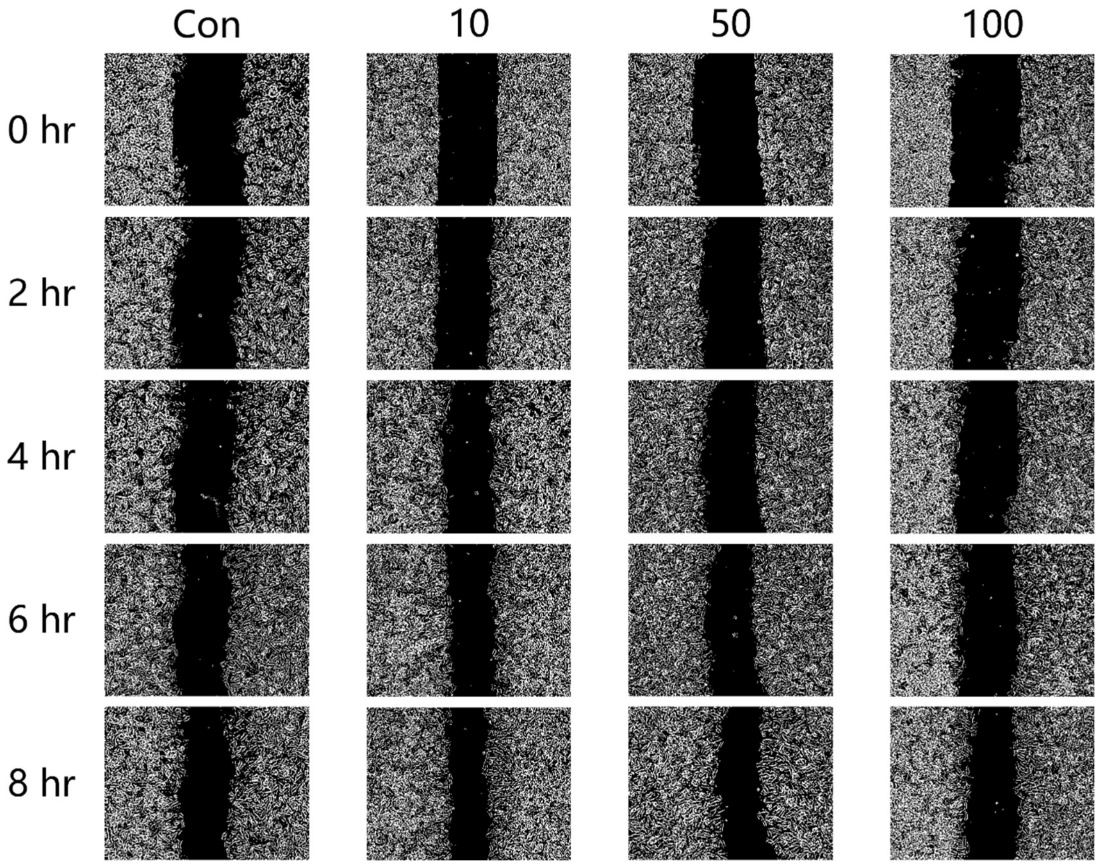
Con
10
50
100
0 hr
2 hr
4 hr
6 hr
8 hr

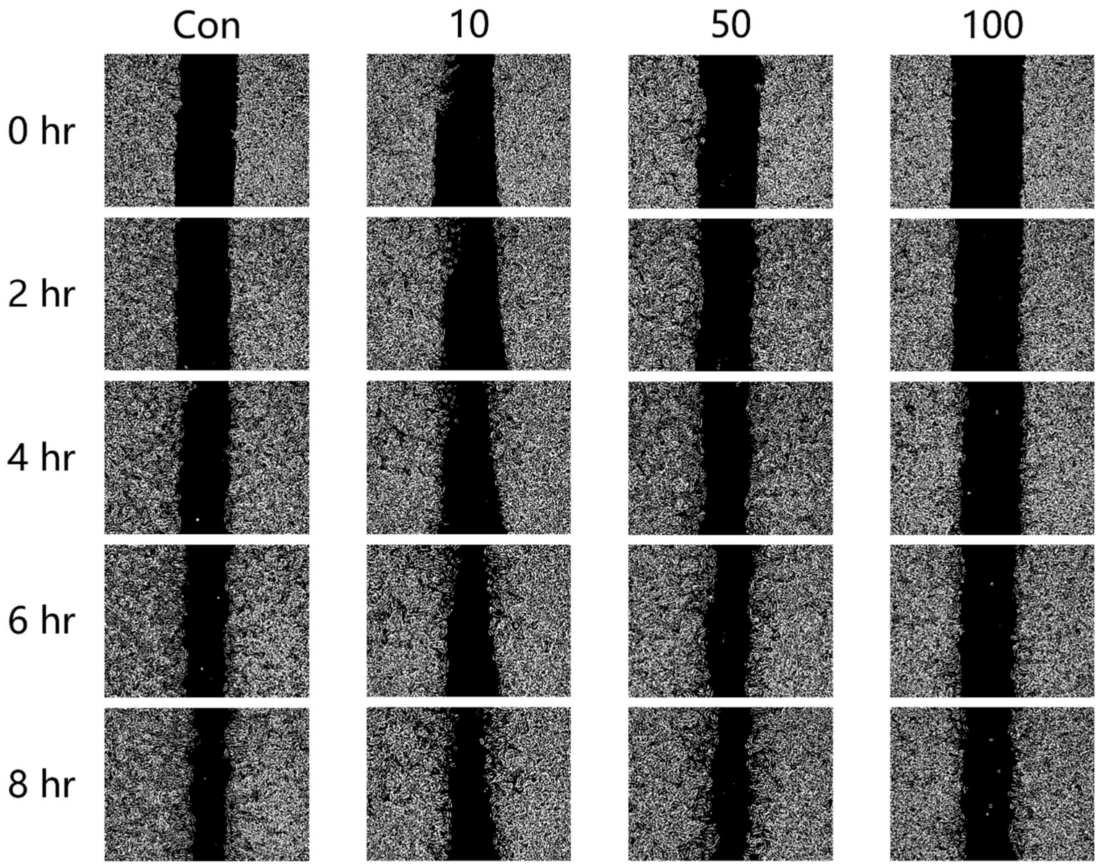
Con
10
50
100
0 hr
2 hr
4 hr
6 hr
8 hr

B. 786-O cells

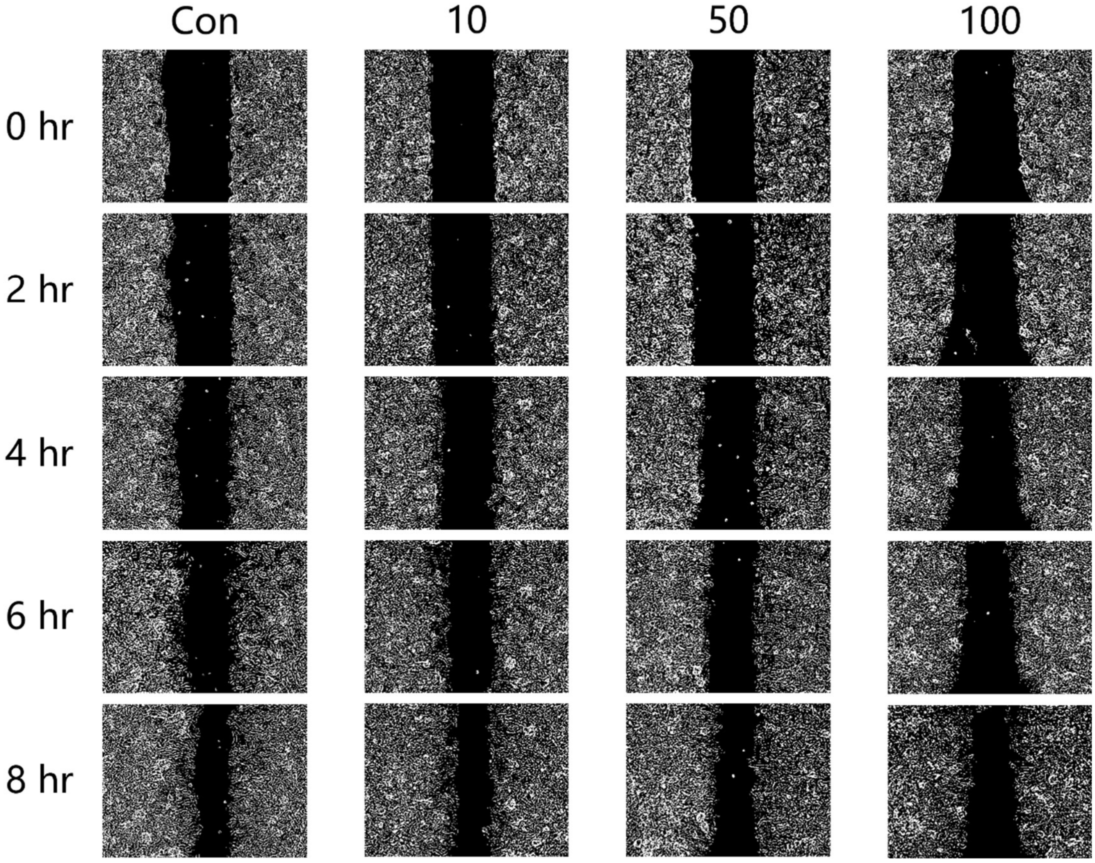
Con
10
50
100
0 hr
2 hr
4 hr
6 hr
8 hr

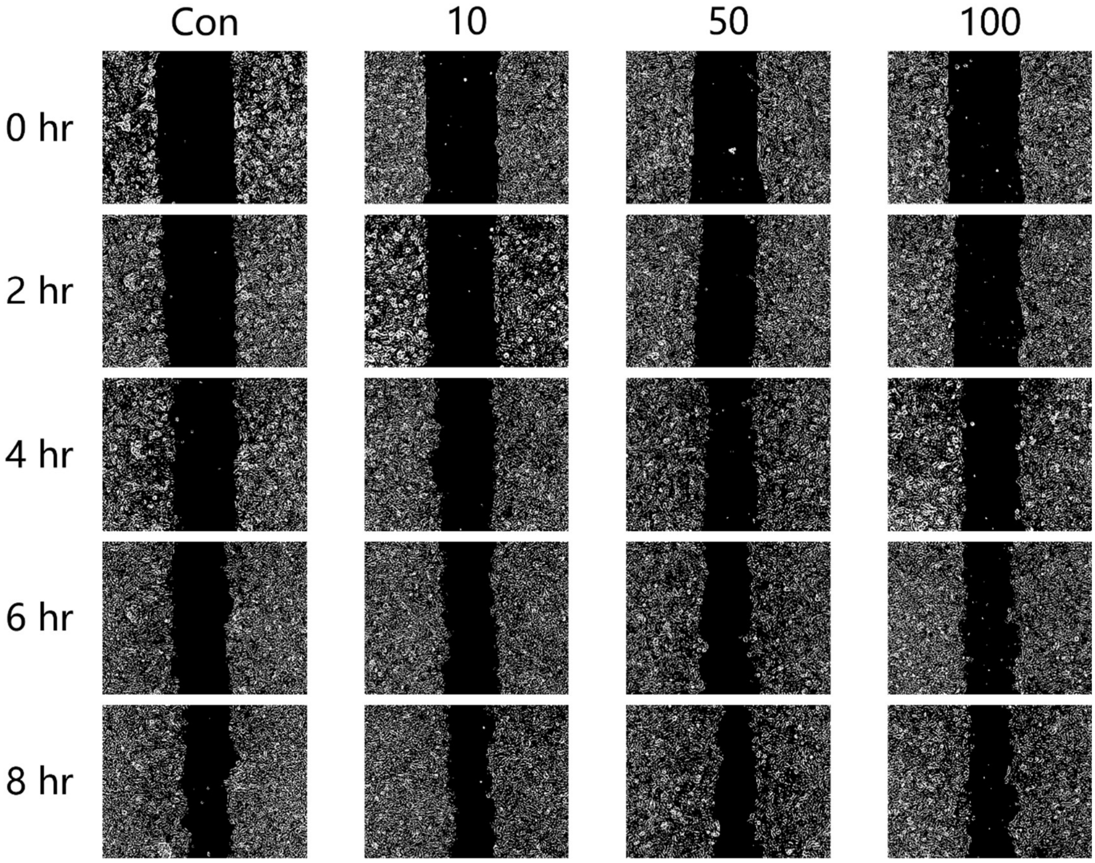
Con
10
50
100
0 hr
2 hr
4 hr
6 hr
8 hr

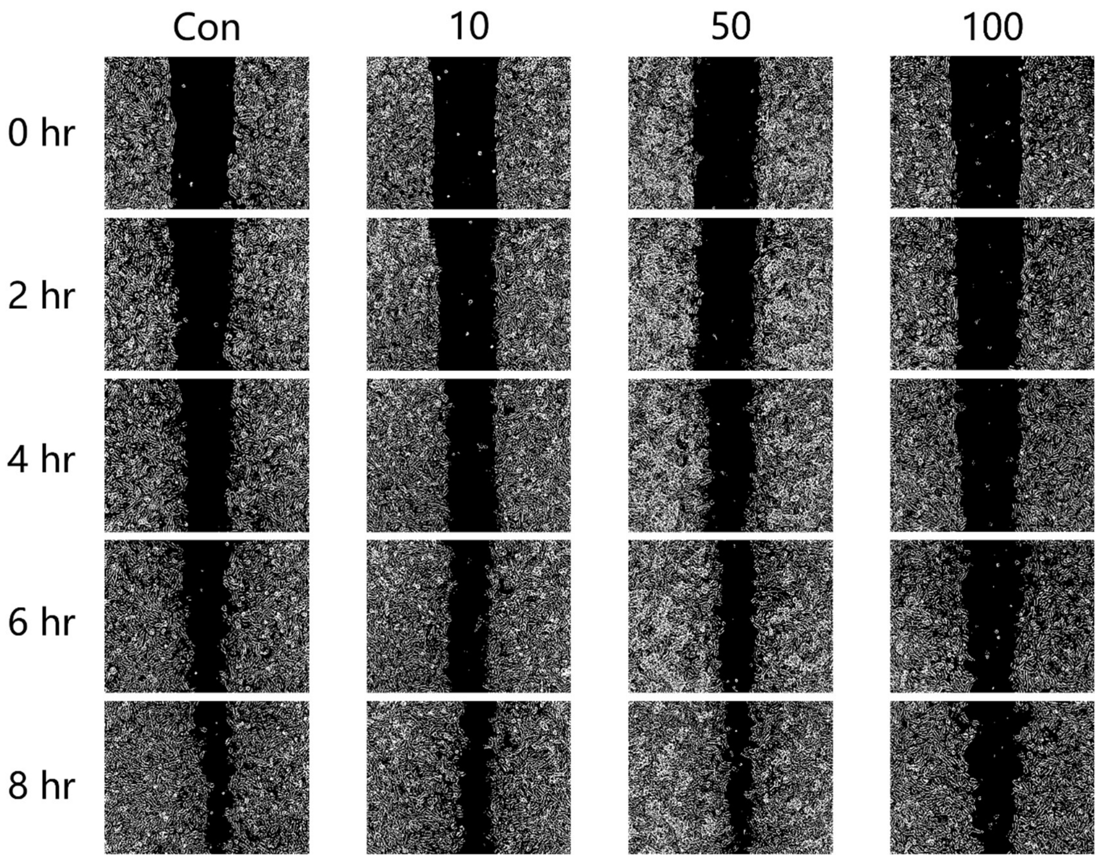

(A, B) The cell monolayer was scratched with a pipette tip and treated with different concentrations of olivomycin A or vesicles as a control in A-498 (A) and 786-O (B) cells. Wound closure was examined at 0, 2, 4, 6, and 8 h after scratching using inverted light microscopy. Images from three independent experiments are shown.
